# Effectiveness of an intermediate care hospital on readmissions, mortality, activities of daily living and use of health care services among hospitalized adults aged 60 years and older—a controlled observational study

**DOI:** 10.1186/s12913-015-1022-x

**Published:** 2015-08-28

**Authors:** Unni Dahl, Aslak Steinsbekk, Roar Johnsen

**Affiliations:** Department of Public Health and General Practice, Norwegian University of Science and Technology, Post box 8905 Medisinsk teknisk forskningssenter, 7491 Trondheim, Norway; Central Norway Health Authority, 7500 Stjørdal, Norway

## Abstract

**Background:**

Intermediate care is a health care model developed to optimize the coordination of health care services and functional independence. In Central Norway, an intermediate care hospital (ICH) was established in a municipality to improve hospital discharge and follow-up among elderly patients with chronic conditions and comprehensive care needs. The aim of this study was to investigate the effectiveness of hospital discharges to a municipality with an ICH compared to discharges to a municipality without an ICH.

**Methods:**

This was a non-randomized controlled observational study of hospitalized patients aged 60 years and older from two municipalities. Patients (*n* = 328) admitted to a general hospital from February 2010 through September 2011 were included in the study and followed for 12 months. The data were analyzed using descriptive statistics, analysis of covariance (ANCOVA) and Cox proportional hazard regression.

**Results:**

Each patient discharged from the general hospital to the municipality with an ICH had a shorter length of stay and used on average 4.2 (*p* = 0.046) fewer hospital days during 1 year compared to patients from the municipality without an ICH. Otherwise, no statistical significant differences were found between the municipalities in terms of hospital readmissions, admissions, mortality, activities of daily living, primary health care utilization or total care days. A post hoc analysis of patients discharged to the ICH compared to the municipality without an ICH, showed that the ICH patients were older and frailer, but the outcome was similar to the main analysis.

**Conclusions:**

Having an ICH in the municipality facilitated shorter length of hospital stay and kept the risk of readmissions, mortality and post-hospitalization care needs at the same level as without an ICH.

## Background

Many health care systems worldwide are facing challenges due to an increasing ageing population [1, 2]. Currently, hospitalized elderly patients with chronic diseases are cared for in a system largely organized for acute care [3, 4]. This approach is often inadequate to meet the specific needs of patients with multiple and complex health conditions. Hence, improving the chain of care to respond to the demands of elderly patients is a key challenge [5–7].

Hospitalizations [8] and discharge [9] can provoke events that negatively affect the patient for a long time after hospitalization. Delayed discharge is associated with acute illness episodes and death [10], and elderly patients discharged from hospital with unmet functional needs have an increased risk of readmissions [11]. It has been documented that 23 % of elderly patients experience an adverse event after discharge such as drug events, therapeutic errors and infections [12].

In response to these health care challenges, interventions and models to improve the coordination of services and safety during hospital discharge have been introduced [13–15]. Intermediate care is one such health care model. It is an umbrella term for a rehabilitation-type arrangement between primary and secondary care that is intended to reduce unnecessary hospital use and optimize functional independence [16]. The term has its origins in the UK [17], however similar models have been developed in several countries to improve and manage changing health care needs [18–20].

Intermediate care services range from nurse-led units to services delivered in the patients’ own home [21]. Due to the different types of intermediate care, summarizing their effectiveness based on evidence is challenging [22]. Studies of intermediate care have shown that the services can enable patients to regain abilities in daily living, decrease readmissions and reduce mortality [19, 23–25]. Still, the reported effects of intermediate care services are inconsistent [21, 26, 27]. In addition to the aforementioned outcomes, addressing the integration of intermediate care into the wider health care system is recommended for further investigation [18, 28, 29].

We previously studied the impact of hospital discharges to an intermediate care hospital (ICH) that was a part of the primary health care system and found that the ICH reduced the length of hospital stays for the municipality inhabitants’, but had only minor influence on the primary health care consumption [30]. However, that study used aggregated, not individual-linked data, from primary and secondary health care. The aim of this study was to investigate the effectiveness of hospital discharges of patients aged 60 years and older to a municipality with an ICH compared to discharges to a municipality without an ICH on readmissions, mortality, activities of daily living (ADL) and health care use during 1 year follow-up.

## Methods

### Study design and ethics

**Fig. 1:**
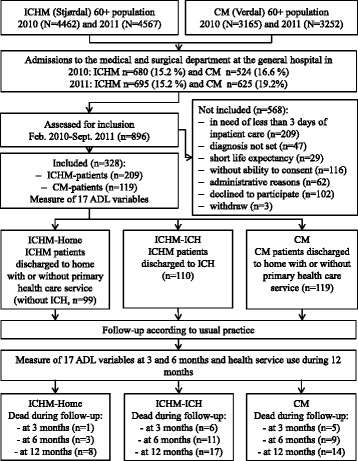
Flow chart of the study. CM = Comparative municipality. ICHM = Intermediate care hospital municipality. ICH = Intermediate care hospital

This was a non-randomized controlled observational study including patients from two municipalities admitted to the same local general hospital in Central Norway (Fig. 1). The intervention municipality established an intermediate care hospital (ICH) in 2007. The other municipality (without an intermediate care hospital) was the control. Hospitalized patients from the two municipalities were included in the study from February 2010 through September 2011. The end of follow-up was October 2012. This evaluation is reported in accordance with the Trend Statement for reporting non-randomized studies of interventions [31].

The Regional Committee for Medical Research Ethics approved the study (2009/1697a) as well as the patient information and consent schemes. The participating municipalities, the ICH and the local general hospital also approved the study. Each participating patient signed a written informed consent at the general hospital prior to inclusion in the study. The study was registered in www.clinicaltrials.gov (Trial number: NCT01706744).

### Setting

The Norwegian health and social care system is primarily a public system. It is organized within two main sectors; the secondary care with hospitals and specialist services on the one hand and primary health care on the other. The hospitals are state owned and organized within health enterprises managed by four regional health authorities. The municipalities are responsible for primary health care services including home care, nursing homes and general practitioners (GPs) [32].

The patients in this study resided in one of two municipalities; the Intermediate care hospital municipality (ICHM) and the Comparative municipality (CM). In 2011 the population size was 21,659 in the ICHM and 14,334 in the CM. Both municipalities are located in the catchment area of a 200-bed general hospital named “Sykehuset Levanger” within the health enterprise “Helse Nord-Trøndelag”.

The hospital admission rates from the ICHM and the CM were stable in the years before the study (2005–2009) and until the end of the follow-up (2010–2012) [30]. In 2010 and 2011 16.6 and 19.2 % of the CM-inhabitants aged 60 years and older were admitted to the medical or surgical department of the general hospital. It was 15.2 % in both years from the ICHM (Fig. 1).

In 2007, the ICHM established the ICH in collaboration with the general hospital and the regional health authorities to offload the hospital with patients ready for discharge and to improve the coordination of services [33]. The ICH is a 12-bed inpatient ward co-located with primary health care services. It is mainly staffed by nurses. Additionally, there are occupational therapists, physiotherapists and a GP during weekdays. The intermediate care is targeted at elderly patients with multiple and chronic conditions and comprehensive care needs who were discharged from the general hospital. When the patients are ready for discharge, they are admitted to the ICH on the same or next day. Discharge is aimed to be earlier when going to the ICH than directly to primary health care services. The ICH-staff emphasize follow-up treatment and enable as many patients as possible to return to their homes. The patients are provided with daily living aids such as walkers and crutches and the staff encourages them to practice activities of daily living (ADL) e.g. walking in the corridor, climbing stairs and socializing with other patients whilst in ICH.

Patients from the ICHM who are not discharged to the ICH and patients from the comparative municipality (CM) were discharged as usual. They were hospitalized until discharge to their homes or until necessary primary health care services were available.

Health professionals’ experiences of patient discharge to municipalities with and without the ICH have been described in a previous qualitative study [34].

### Participants

The inclusion and exclusion criteria were selected to establish a group of patients who were eligible for an ICH stay regardless of whether an ICH was available. The inclusion criteria were patients living in the ICHM or the CM, aged 60 years and older, admitted from their own homes to the medical or surgical department at the general hospital, assessed to be in need of in-patient care for at least 3 days after completion of the hospital diagnostics and the initial treatment and expected to return home after in-patient care. The exclusion criteria were patients without the ability to consent (due to dementia or other medical conditions) and short life expectancy.

The main comparison groups were patients from the ICHM and CM. However, the ICHM patients comprised two groups; those discharged directly from the hospital to the ICH and those who were discharged directly to home with or without the need for primary health care services.

### Data collection

Nurses included patients during the first days of hospitalization at the general hospital (index stay). Inclusion was performed before the hospital physicians had decided where the patients should be discharged. The data were collected at the index stay and during 1 year of follow-up after hospital discharge or until death.

Information on hospital stays were collected from the general hospital’s register, the university hospital’s register and from the national register (Norwegian Patient Register, NPR). Stays at specialized rehabilitation centers were collected directly from the rehabilitation institutions in Central Norway and supplemented by national data from the NPR.

Data on the use of primary health care services were collected from the municipalities’ own registers. The ICH stay data were collected from the ICH and subsequently added to the institutional primary health care stays in the ICHM.

Nurses in the general hospital and in the primary health care services were selected to measure and record 17 ADL variables at index hospital stay and at 3 and 6 months follow-up. These ADLs have been compulsorily and routinely recorded for individuals who request or are in need of primary health care services in all Norwegian municipalities since 2006 (IPLOS) [35, 36]. IPLOS is a register-based information system, used in other Norwegian studies [37, 38], that characterizes patient dependencies by the following variables: eating, dressing, personal hygiene, using the toilet, indoor and outdoor mobility, cooking, housekeeping, shopping, maintaining own health, communication, social interaction, daily decision taking, memory, behavioural control, sight and hearing. The ADL variables are used to assess the patients’ need for assistance, and trained personnel perform the scoring. Each ADL is measured on a scale from one to five. A score of one means no disability; two indicates some functional challenges while three is given when the patient is in need of assistance. A score of four is used for an increased need of assistance and five means extensive need of assistance. In a hospital setting, patients do not perform all activities even if they are capable of doing so, e.g. housekeeping and cooking. Nevertheless, all patients in this study were scored according to their potential capacity to perform the activities [37]. The functional status was calculated as the mean value of all 17 ADL variables.

Publicly available population data was collected from Statistics Norway [39].

### Outcome measures

The outcomes were measured 3 months and 1 year from index hospital admission except for the proportion of patients with readmissions within 30 days. The functional status was measured at 3 and 6 months from the index hospital admission. Hospitalization and rehabilitation stays were taken from a national data register. Hospital admissions, hospital days and length of hospital stay included the patients’ index stay. Hospital admissions were calculated as any in-patient stay (acute and elective) as well as the number of acute admissions after index hospital discharge was counted. A readmission was defined as any acute hospital admission within 30 days from a previous discharge [40] regardless if the readmission occurred during the ICH stay. For readmission incidents within 30 days during 1 year, the first subsequent readmission was counted. It then became a new admission from which a further readmission might occur [41]. Total days with institutional care comprised all in-patient stays, i.e., hospital days, institutional primary health care, ICH, and specialized rehabilitation. Hour-based primary health care services included home care nursing, practical assistance at home, day-center visits, and other types of support to persons living at home. Institutional primary health care were short- and long-term stays in nursing homes. The ICH stays were included in the institutional primary health care in the ICHM. Mortality was measured as the number of deceased patients during 1 year of follow-up after the index hospital discharge, and number of days until death.

### Statistical analysis

A comparison of the outcomes for the two municipalities (ICHM and CM) was the primary analysis. Descriptive analyses were used to describe the samples. For categorical variables, Pearson’s chi-square tests were used to identify any differences in proportions between the municipalities; Student’s *t*-test was used for continuous variables. Between-group differences for readmissions, health care use and functional status were analyzed with analysis of covariance (ANCOVA). The Cox proportional hazard regression was used to estimate adjusted hazard ratios (HRs) for death. Departure from the proportional hazard assumption was evaluated by graphical procedures (log-log plots).

Patient characteristics are predictors of the use of health care services. Therefore, age, gender, number of diagnoses, functional status and having primary health care (yes/no) at the index stay were included as adjustment variables. A post hoc analysis was conducted to examine the group of patients in the ICHM discharged directly to the ICH (ICHM-ICH) to those discharged to the CM. A significance level of 5 % (*p* < 0.05) was chosen. The analysis was performed using SPSS 21.0 for Windows (IBM Corp., Armonk, NY).

## Results

A total of 896 patients aged 60 years and older from the two municipalities were assessed for inclusion by the department nurses; 463 did not meet the inclusion criteria, 102 declined to participate and three patients withdrew after inclusion (Fig. 1). Consequently 328 patients were included. Two hundred and nine patients resided in the Intermediate care hospital municipality (ICHM-group). Of these, 110 were discharged directly to a stay at the intermediate care hospital (ICHM-ICH) and 99 were discharged to home (ICHM-Home). There were 119 patients discharged, as per usual practice, from the hospital to the Comparative municipality (CM-group).

### Baseline characteristics

At baseline, the ICHM and CM patients were comparable with respect to gender, number of diagnoses, functional status, proportion having acute admissions and proportion receiving primary health care (Table 1). The CM-group was younger (mean age, 72.9 vs 75.5 years, *p* = 0.007). The average diagnosis related group (DRG) weight was higher (2.24 vs 1.76 points, *p* = 0.007) than in the ICHM-group. The index hospital stay was longer in the CM than in the ICHM (8.9 vs 5.5 days, *p* < 0.001).

**Table 1:** Baseline characteristics. Characteristics of patients aged 60 years and older from the CM^a^ and the ICHM^a^ at index hospital stay and those in the ICHM discharged directly to the ICH (ICHM-ICH)^a^

	CM - group	ICHM - group		*p*-value	*p*-value
	Total	Total	ICHM-ICH	CM vs ICHM	CM vs ICHM-ICH
Cases (N)	119	209	110		
Female (n (%))	59 (49.6 %)	118 (56.5 %)	68 (61.8 %)	0.229	0.063
Age (mean (SD))	72.9 (8.2)	75.5 (8.7)	78.2 (8.6)	0.007	<0.001
-Age group 60 (n (%))	47 (39.5 %)	57 (27.3 %)	19 (17.3 %)	0.059	<0.001
-Age group 70 (n (%))	44 (37.0 %)	81 (38.8 %)	39 (35.5 %)		
-Age group 80 (n (%))	26 (21.8 %)	60 (28.7 %)	42 (38.2 %)		
-Age group 90 (n (%))	2 (1.7 %)	11 (5.3 %)	10 (9.1 %)		
No. of diagnoses (mean (SD))	3.9 (2.2)	4.2 (2.0)	4.5 (1.9)	0.238	0.048
DRG weight (mean (SD))	2.24 (1.63)	1.76 (1.38)	1.85 (1.40)	0.007	0.057
Acute hospital admission (n (%))	84 (70.6 %)	163 (78.0 %)	87 (79.1 %)	0.135	0.139
Length of index hospital stay [days] (mean (SD))	8.9 (7.3)	5.5 (4.4)	5.0 (4.8)	<0.001	<0.001
Functional status at inclusion (mean (SD))	1.95 (0.82)	1.95 (0.74)	2.20 (0.73)	0.976	0.016
Primary health care services at admission [yes/no] (n (%))	41 (34.5 %)	75 (35.9 %)	52 (47.3 %)	0.794	0.048

^a^ *CM* Comparative municipality, *ICHM* Intermediate care hospital municipality, *ICHM-ICH* Patients in the ICHM discharged directly to the ICH (Intermediate care hospital)

### The ICHM-group versus the CM-group

Each patient from the CM spent statistically significantly more days in the hospital during 1 year compared to patients from the ICHM (adj. mean difference within 3 months was 3.81 days, *p* = 0.001; during 12 months it was 4.19 days, *p* = 0.046) (Table 2). During 1 year of follow-up, the average length of the hospital stay was 2.39 more days per patient in the CM (*p* < 0.001).

**Table 2:** Comparison of the ICHM-group with the CM-group. Comparison of the ICHM-group^a^ with the CM-group^a^ on use of health care services and functional status during 1 year after hospitalization for patients aged 60 years and older. (CM: 119 patients. ICHM: 209 patients)^d^

Variable	Municipality	Crude mean (SD)	Adj. mean (95 % CI)	Adj. mean diff. (95 % CI)	*p*-value
Use of hospital care					
Mean number of hospital admissions during 3 months	CM	1.55 (1.01)	1.52 (1.34 to 1.70)	−0.09 ( −0.31 to 0.14)	0.446
	ICHM	1.58 (1.04)	1.61 (1.47 to 1.74)		
Mean number of hospital admissions during 1 year	CM	2.25 (2.06)	2.18 (1.79 to 2.56)	−0.41 ( −0.89 to 0.07)	0.092
	ICHM	2.54 (2.26)	2.59 (2.30 to 2.87)		
- Acute admissions during 3 months	CM	0.40 (0.87)	0.38 (0.23 to 0.52)	−0.06 ( −0.25 to 0.12)	0.481
	ICHM	0.43 (0.80)	0.44 (0.33 to 0.55)		
- Acute admissions during 1 year	CM	0.92 (1.97)	0.86 (0.52 to 1.19)	−0.37 ( −0.80 to 0.05)	0.085
	ICHM	1.19 (1.92)	1.23 (0.98 to 1.48)		
Proportion of patients with readmissions within 30 days	CM	0.15 (0.36)	0.14 (0.08 to 0.20)	−0.01 ( −0.09 to 0.08)	0.901
	ICHM	0.14 (0.35)	0.15 (0.10 to 0.19)		
Mean number of readmission incidents (within 30 days) during 1 year	CM	0.44 (1.42)	0.39 (0.15 to 0.63)	−0.20 ( −0.50 to 0.10)	0.195
	ICHM	0.56 (1.29)	0.59 (0.41 to 0.77)		
Mean number of hospital days during 3 months	CM	12.67 (11.93)	12.58 (10.83 to 14.33)	3.81 (1.61 to 6.01)	0.001
	ICHM	8.67 (8.82)	8.77 (7.46 to 10.08)		
Mean number of hospital days during 1 year	CM	18.07 (23.85)	17.67 (14.41 to 20.94)	4.19 (0.08 to 8.31)	0.046
	ICHM	13.15 (14.82)	13.48 (11.03 to 15.93)		
Mean length of hospital stay during 1 year (days)	CM	7.71 (5.88)	7.74 (6.88 to 8.60)	2.39 (1.31 to 3.47)	<0.001
	ICHM	5.35 (4.31)	5.36 (4.71 to 6.00)		
Total care days					
Mean number of total days with institutional care during 1 year^b^	CM	32.40 (44.05)	32.57 (25.05 to 40.08)	1.99 ( −7.46 to 11.45)	0.679
	ICHM	30.41 (42.24)	30.57 (24.94 to 36.21)		
Use of primary health care					
Mean number of hour-based primary health care service during 3 months	CM	25.72 (56.60)	26.38 (17.13 to 35.63)	2.49 ( −9.16 to 14.13)	0.675
	ICHM	24.03 (57.95)	23.89 (16.95 to 30.83)		
Mean number of hour-based primary health care service during 1 year	CM	87.36 (175.89)	88.05 (56.28 to 119.82)	−0.76 ( −40.75 to 39.23)	0.970
	ICHM	88.36 (220.09)	88.81 (64.98 to 112.64)		
Mean number of days in institutional primary health care during 3 months	CM	6.27 (18.41)	6.80 (4.49 to 9.12)	0.51 ( −2.41 to 3.42)	0.732
	ICHM	6.54 (10.13)	6.29 (4.56 to 8.03)		
Mean number of days in institutional primary health care during 1 year	CM	11.49 (33.04)	12.19 (6.23 to 18.15)	−1.77 ( −9.27 to 5.73)	0.643
	ICHM	14.24 (34.37)	13.96 (9.49 to 18.43)		
- Mean number of days in nursing home (long-term)	CM	1.80 (13.15)	1.90 ( −1.65 to 5.45)	−1.10 ( −5.57 to 3.37)	0.627
	ICHM	3.03 (22.07)	3.00 (0.34 to 5.66)		
- Mean number of days in ICH	CM	0.00 (0.00)	0.15 ( −1.55 to 1.85)	−8.53 ( −10.67 to −6.39)	<0.001
	ICHM	8.72 (12.07)	8.68 (7.41 to 9.96)		
ADL^c^					
Mean functional status at 3 months	CM	1.51 (0.52)	1.53 (1.46 to 1.60)	−0.01 ( −0.11 to 0.08)	0.762
	ICHM	1.55 (0.48)	1.54 (1.49 to 1.60)		
Mean functional status at 6 months	CM	1.52 (0.56)	1.53 (1.45 to 1.61)	0.00 ( −0.11 to 0.10)	0.945
	ICHM	1.54 (0.49)	1.53 (1.47 to 1.59)		

Analysis with ANCOVA adjusted for age, gender, number of diagnoses at index hospital stay, functional status at index hospital stay and having primary health care services at index hospital stay (yes/no)

^a^ *CM* Comparative municipality, *ICHM* Intermediate care hospital municipality, *ICH* Intermediate care hospital

^b^Mean number of total days with institutional care = In-patient stays at hospital, primary health care, ICH and specialized rehabilitation

^c^ADL = Activities of daily living assessed with IPLOS (a national registration system with 17 ADL variables)

^d^At index hospital stay patients with missing functional status were 1 in the CM and 1 in the ICHM. At 3 months the number of patients was six and seven, respectively and at 6 months it was eight and 18

Table 2 shows no statistical significant differences between the municipalities in the number of hospital admissions (including acute admissions) or the number of readmission incidents (within 30 days) during 1 year. Moreover, no differences were found for the proportion of patients with readmissions within 30 days. The results showed no differences in hour-based primary health care, days spent in institutional primary health care (including long-term stays in nursing homes), or in functional status. As a sensitivity analysis, the non-measured functional status for those who died within 3 and 6 months was replaced by a value of 5 (poorest functionality). This caused a small increase in the mean score of the functional status at 3 and 6 months. No difference between the groups remained. Interaction between adjustment variables (covariates) and group was checked and was not significant. In general, the adjustments decreased the hospital use in the CM (except for the length of hospital stay) and increased the use in the ICHM. For primary health care, the adjustments increased the use in the CM but decreased in the ICHM (except for hour-based primary health care during 1 year).

**Fig. 2:**
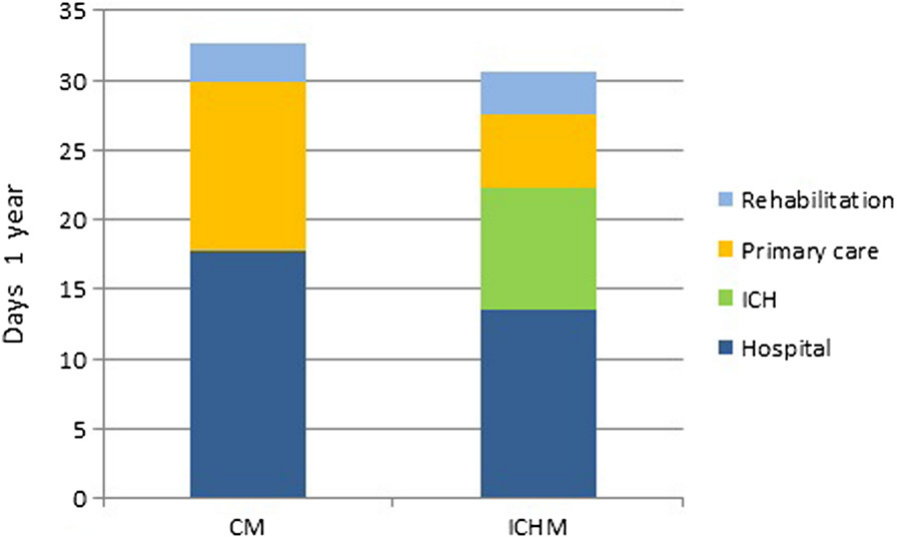
Total care days during 1 year. The average number of days in hospital, institutional primary health care, the ICH and specialized rehabilitation for each patient aged 60 years and older in the ICHM and the CM during 1 year. Analysis with ANCOVA adjusted for age, gender, number of diagnoses at index hospital stay, functional status at index hospital stay, and having primary health care services at index stay (yes/no). *CM*  Comparative municipality. *ICHM*  Intermediate care hospital municipality. *ICH*  Intermediate care hospital

Comparing the total number of days with institutional care by adding up all in-patient stays at hospital, primary health care, ICH and specialized rehabilitation during 1 year showed no difference between the municipalities (adj. mean CM = 32.6, ICHM = 30.6 days per patient). Of the days spent in primary health care institutions in the ICHM, 8.7 days per patient was spent in the ICH (Table 2 and Fig. 2).

Regarding mortality, no difference was observed between patients from the CM (11.8 %) and the patients from the ICHM (12.0 %) (Table 3). There was no evidence of departure from the proportional hazards assumption.

**Table 3:** Hazard ratios (HRs) for death 1 year after hospitalization for persons 60 years and older

Municipality	Persondays	Deaths, n (%)	Crude HR^b^	Adjusted HR^c^	95 % CI^c^	*p*-value^c^
CM^a^	40,429	14 (11.8)		1		
ICHM^a^	71,321	25 (12.0)	1.01	0.93	0.48–1.81	0.840
ICHM-ICH^a^	36,420	17 (15.5)	1.34	1.01	0.49–2.08	0.981

^a^ *CM* Comparative municipality, *ICHM* Intermediate care hospital municipality, *ICHM-ICH* Patients in the ICHM discharged directly to the ICH (Intermediate care hospital)

^b^Unadjusted

^c^Adjusted for age, gender, number of diagnoses at index hospital stay, functional status at index hospital stay and having primary health care services at index stay (yes/no)

### Post hoc analysis

At baseline, the patients discharged from hospital directly to the ICH (ICHM-ICH) were 5.3 years older than the CM-group (Table 1). Gender, DRG weight and proportion with acute admissions were comparable. The ICHM-ICH group had a higher mean number of diagnoses, worse functional status, and a higher proportion received primary health care prior to hospitalization indicating that this group was sicker and had poorer functionality, i.e., was more frail. The average index hospital stay was shorter in the ICHM-ICH group. The average length of the ICH stay after index hospital discharge was 8.3 days (data not shown in tables).

The patients in the ICHM who were discharged directly to home (ICHM-Home, data not shown in tables) had a lower DRG weight at baseline (1.65 points vs 2.24, *p* = 0.004), better functional status (1.68 vs 1.95, *p* = 0.006) and shorter length of index hospital stay (6.1 vs 8.9 days, *p* < 0.001) compared to the CM-group.

The post hoc analysis of the outcomes comparing the CM and the ICHM-ICH group did not substantially deviate from the results of the comparison on the municipality level presented above (Table 4). The main difference was that patients in the CM-group spent 3.75 more days (*p* = 0.013) in the hospital within 3 months than the ICHM-ICH group. During 1 year of follow-up, the average length of hospital stay was 2.33 more days per patient in the CM-group (*p* = 0.002).

**Table 4:** Post hoc analysis. Comparison of the ICHM-ICH group with the CM-group. Comparison of the ICHM-ICH^a^ with the CM-group^a^ on use of health care services and functional status during one year after hospitalization for patients aged 60 years and older. (CM: 119 patients. ICHM-ICH: 110 patients)^d^

Variable	Municipality	Crude mean (SD)	Adj. mean (95 % CI)	Adj. mean diff. (95 % CI)	*p*-value
Use of hospital care					
Mean number of hospital admissions during 3 months	CM	1.55 (1.01)	1.53 (1.35 to 1.72)	−0.05 ( −0.32 to 0.22)	0.720
	ICHM-ICH	1.56 (1.03)	1.58 (1.39 to 1.78)		
Mean number of hospital admissions during 1 year	CM	2.25 (2.06)	2.26 (1.88 to 2.63)	−0.19 ( −0.74 to 0.37)	0.512
	ICHM-ICH	2.45 (2.11)	2.44 (2.05 to 2.83)		
- Acute admissions during 3 months	CM	0.40 (0.87)	0.41 (0.25 to 0.56)	−0.02 ( −0.25 to 0.20)	0.839
	ICHM-ICH	0.44 (0.81)	0.43 (0.27 to 0.59)		
- Acute admissions during 1 year	CM	0.92 (1.97)	0.96 (0.61 to 1.30)	−0.20 ( −0.70 to 0.31)	0.449
	ICHM-ICH	1.20 (1.85)	1.15 (0.80 to 1.51)		
Proportion of patients with readmissions within 30 days	CM	0.15 (0.36)	0.15 (0.09 to 0.22)	0.03 ( −0.07 to 0.12)	0.586
	ICHM-ICH	0.14 (0.34)	0.13 (0.06 to 0.19)		
Mean number of readmission incidents (within 30 days) during 1 year	CM	0.44 (1.42)	0.43 (0.18 to 0.69)	−0.16 ( −0.54 to 0.22)	0.419
	ICHM-ICH	0.59 (1.36)	0.59 (0.32 to 0.86)		
Mean number of hospital days during 3 months	CM	12.67 (11.93)	12.62 (10.62 to 14.62)	3.75 (0.79 to 6.71)	0.013
	ICHM-ICH	8.75 (10.47)	8.87 (6.80 to 10.95)		
Mean number of hospital days during 1 year	CM	18.07 (23.85)	18.08 (14.41 to 21.73)	4.99 ( −0.43 to 10.42)	0.071
	ICHM-ICH	12.98 (15.47)	13.08 (9.28 to 16.88)		
Mean length of hospital stay during 1 year (days)	CM	7.71 (5.88)	7.76 (6.77 to 8.75)	2.33 (0.86 to 3.80)	0.002
	ICHM-ICH	5.45 (5.36)	5.43 (4.41 to 6.46)		
Total care days					
Mean number of total days with institutional care during 1 year^b^	CM	32.40 (44.05)	34.58 (25.94 to 43.23)	−0.50 ( −13.32 to 12.32)	0.939
	ICHM-ICH	37.18 (51.10)	35.08 (26.11 to 44.06)		
Use of primary health care					
Mean number of hour-based primary health care service during 3 months	CM	25.72 (56.60)	30.61 (20.21 to 41.00)	0.34 ( −15.06 to 15.74)	0.966
	ICHM-ICH	35.28 (66.77)	30.27 (19.48 to 41.05)		
Mean number of hour-based primary health care service during 1 year	CM	87.36 (175.89)	105.39 (71.55 to 139.24)	0.16 ( −50.01 to 50.32)	0.995
	ICHM-ICH	123.79 (247.55)	105.24 (70.11 to 140.36)		
Mean number of days in institutional primary health care during 3 months	CM	6.27 (18.41)	7.81 (5.08 to 10.53)	−2.16 ( −6.21 to 1.88)	0.293
	ICHM-ICH	11.56 (11.36)	9.97 (7.14 to 12.80)		
Mean number of days in institutional primary health care during 1 year	CM	11.49 (33.04)	13.89 (6.89 to 20.89)	−5.52 ( −15.89 to 4.86)	0.296
	ICHM-ICH	21.87 (43.24)	19.40 (12.14 to 26.67)		
- Mean number of days in nursing home (long-term)	CM	1.80 (13.15)	2.02 ( −2.27 to 6.31)	−3.10 ( −9.47 to 3.26)	0.338
	ICHM-ICH	5.35 (29.99)	5.12 (0.67 to 9.58)		
- Mean number of days in ICH	CM	0.00 (0.00)	0.08 ( −1.39 to 1.55)		
	ICHM-ICH	12.95 (11.25)	12.86 (11.34 to 14.39)	−12.79 ( −14.96 to −10.61)	<0.001
ADL^c^					
Mean functional status at 3 months	CM	1.51 (0.52)	1.59 (1.50 to 1.67)	−0.05 ( −0.17 to 0.07)	0.410
	ICHM-ICH	1.72 (0.55)	1.64 (1.55 to 1.72)		
Mean functional status at 6 months	CM	1.52 (0.56)	1.57 (1.48 to 1.66)	−0.05 ( −0.19 to 0.08)	0.444
	ICHM-ICH	1.69 (0.53)	1.63 (1.53 to 1.72)		

Analysis with ANCOVA adjusted for age, gender, number of diagnoses at index hospital stay, functional status at index hospital stay and having primary health care services at index hospital stay (yes/no)

^a^ *CM* Comparative municipality, *ICHM-ICH* Patients in the ICHM discharged directly to the ICH (Intermediate care hospital)

^b^Mean number of total days with institutional care = In-patient stays at hospital, primary health care, ICH and specialized rehabilitation

^c^ADL = Activities of daily living assessed with IPLOS (a national registration system with 17 ADL variables)

^d^At index hospital stay patients with missing functional status were 1 in the CM and 0 in the ICHM-ICH. At 3 months the number of patients was six and three, respectively and at 6 months it was eight and 11

There were no statistical significant differences in the number of hospital admissions, readmissions, total days with institutional care, received primary health care, functional status or mortality between the groups (Tables 3 and 4).

## Discussion

The main result from this study was that patients discharged from the hospital to the municipality with an ICH (the ICHM) had 4.2 fewer hospitalization days and shorter length of stays during 12 months of follow-up than the municipality without an ICH (the CM). No statistical significant differences in the other outcomes were observed. A post hoc analysis of those that were discharged directly to the ICH compared to the CM, gave similar findings as the main analysis.

### Integrating intermediate care in the chain of care

The main finding here is comparable to a previous cohort study of all hospitalized patients aged 60 years and older residing in the ICHM and the CM from 2008 through 2012 [30]. In both studies, the patients in the ICHM used fewer hospital days.

Preventing prolonged hospital stays, strengthening primary health care and improving coordination of services are prominent goals of the Norwegian health policy [20]. The first goal was met in this study because patients in the municipality with the ICH spent fewer days in hospital. Nevertheless, studies from different countries have described how difficult it is to move patients smoothly through the chain of care due to collaboration challenges [42, 43] and lack of integration of intermediate care with the whole care system [18, 28]. Findings in the current and a previous qualitative study in the ICHM and CM [34] are, however, more in line with recommendations to improve coordination of discharges for patients with complex care needs [6, 13]. It seems reasonable to explain the lower use of hospital days in the ICHM by the health personnel’s shared goal of preventing prolonged hospital stays by facilitating early and timely hospital discharges to the ICH [34].

In accordance with the target group of the ICH (elderly patients in need of follow-up care), this study confirms that the patients discharged directly to the ICH were the oldest with the poorest functionality, highest number of diagnoses and most in need of primary health care. One could assume that this group of patients, that was more frail than the average of the ICHM-group would need longer hospital stays. Still, the number of days in hospital for the patients discharged directly to the ICH was similar to patients in the ICHM-group. This suggested that the ICH helped reduce the hospital utilization for frail patients.

During the 1 year follow-up, the number of days in hospital was 31 % higher in the CM (4.2 days) than in the ICHM. The average length of hospital stays per patient in the CM was 45 % (2.4 days) longer. The number of days spent in institutional primary health care was similar in the municipalities even when this included the stays in the ICH, which comprised 61 % of all primary health care days in the ICHM. Also, utilization of hour-based care was similar in the municipalities. Hence, early discharges did not influence the utilization of primary health care services in the ICHM compared to the CM. This confirms previous qualitative findings in which ICH was used as a discharge unit in the chain of care [34]. The ICH facilitated early hospital discharge for patients who were in need of further institutional care, reduced the pressure on hospital inpatient services and prevented prolonged hospital stays.

### Readmissions, mortality and activities of daily living

Decreases in the length of hospital stay over the last decade [2] have raised concerns that early discharges may increase readmissions and mortality rates [44, 45]. This would imply that elderly people in the ICHM could be at risk because they were discharged earlier. In contrast to previous studies indicating that intermediate care may increase [26] or decrease readmissions [27], reduce mortality [24] or enable patients to regain abilities in daily living [19, 23], this study found no statistical significant differences between the municipalities. This was true for readmissions, mortality and ADL scores. These results alleviate the concern that the shorter length of hospitalizaton in elderly patients in a municipality with a discharge unit such as the ICH will lead to an increased risk of readmissions or deaths. This is supported by findings in other studies [46]. Indeed, a study of hospital discharges of patients aged 65 and older to skilled nursing facilities concluded that a 1-day reduction in length of hospital stay was not consistently associated with a higher rate of 30-day readmission [47].

### Strengths and limitations

The strengths of this study were the inclusion of participants from two municipalities that were comparable on most baseline characteristics, the adjustment of the analyses for the differences in the groups, the collection of data from reliable electronic registers and the linkage of data on an individual level. The patients included in this study seem to be representative of all patients discharged to the ICH: For all patients from the ICHM aged 60 years and older who were discharged from the general hospital to the ICH from 2008 to 2011 [30], the average age was 79 years and 59 % were female. In the present study, the average age was 78.2 years and 61.8 % were female.

The major limitation was the study design, which was chosen because the ICH was already in operation. Hence, randomizing patients to the ICH or not was omitted due to both practical and ethical reasons. Additionally, we were interested in how the ICH operated within the primary health care service, i.e. the municipality level.

It should also be noted that the patients who lived alone were not registered and thus not adjusted for. Some patients could not consent due to their condition (e.g. strong analgesics or cognitive impairment). Moreover, the ICH model may be different in other settings which limits the generalizability. Cost analysis of alternative care models should be considered in future research.

## Conclusions

The results show that patients from the municipality with access to an ICH used fewer hospital days and had a similar primary health care utilization among patients aged 60 years and older as patients from the municipality without an ICH. Having an ICH facilitated early hospital discharge for the patients and kept the risk of readmissions, mortality and the abilities to perform activities of daily living at the same level as without an ICH.

## Abbreviations

ADL: Activities of daily living
CM: Comparative municipality (Verdal municipality)
ICH: Intermediate care hospital
ICHM: Intermediate care hospital municipality (Stjørdal municipality)
ICHM-Home: The patients in the ICHM who were discharged directly to home
ICHM-ICH: The patients in the ICHM who were discharged directly to the ICH
